# Study on the Fast Search Planning Problem of Lost Targets for Maritime Emergency Response Based on an Improved Adaptive Immunogenetic Algorithm

**DOI:** 10.3390/s24123904

**Published:** 2024-06-17

**Authors:** Tianyue Yu, Yasheng Zhang, Jie Yang

**Affiliations:** 1 Graduate School, Space Engineering University, Beijing 101400, China; 18810975861@163.com; 2 Unit 63798 of the People’s Liberation Army, Xichang 615600, China; 17602842616@163.com

**Keywords:** moving target search, mission planning, adaptive immunogenetic algorithm

## Abstract

This study investigates the problem of rapid search planning for moving targets in maritime emergencies using an improved adaptive immune genetic algorithm. Given the complexity and uncertainty inherent in searching for moving targets in maritime emergency situations, a task planning method based on the improved adaptive immunogenetic algorithm (IAIGA) is proposed to enhance search efficiency and accuracy. This method utilizes a priori information to construct the potential regions of the target and the distribution probability within each region. It establishes a “prediction-scheduling” search strategy model, planning a rapid search task for disconnected targets based on overlapping probability through the IAIGA. By incorporating an immune mechanism, the algorithm enhances its global search capability and robustness. Additionally, the adaptive strategy enables dynamic adjustment of the algorithm’s parameters to accommodate varying search scenarios. The experimental results demonstrate that the proposed IAIGA significantly outperforms traditional methods, providing higher search speeds and more accurate search results in the context of maritime emergency response. These findings offer effective technical support for maritime emergency operations.

## 1. Introduction

The search for lost moving targets at sea is an essential component of emergency response missions. These missions encompass non-confrontational tasks such as marine rescue and plankton monitoring, as well as confrontational tasks such as maritime anti-smuggling operations. The loss of target connection, i.e., the loss of communication with mobile targets on the sea surface, complicates the determination of their specific positions by electronic reconnaissance satellites, thereby increasing the search difficulty. When target communication is lost and a priori information is limited, the complexity of the search escalates over time, necessitating extremely rapid search operations. Early research on moving target searches primarily focused on scenarios with known target distribution patterns. The goal was to quickly locate the target by efficiently scheduling available resources to optimize the allocation of search assets and trajectory planning. This allocation scheme is particularly suited for unmanned aerial vehicles (UAVs) [1,2,3], robots [4], airships [5], and other reconnaissance resources whose trajectories can be planned. However, the use of manned resources in search missions is constrained by manpower, speed, and endurance. In contrast, satellites offer a larger observable range and imaging width, free from geographical boundaries, making them ideal for mobile target search missions over extensive sea areas. Imaging satellites are categorized based on their orbital altitudes into high-orbit and low-orbit satellites. Low-orbit imaging satellites, in particular, are more numerous and can be networked to increase the frequency of visits to the target area. This networking capability reduces the satellite revisit time, thereby compensating for the inherent limitations of the discrete observation windows in single-satellite systems [6]. However, imaging satellites are constrained by various factors such as illumination, solar incidence angle, and side-swing angle, which affect their mission performance. Additionally, the detection range of a single mission is relatively small. Current satellite mission scheduling studies lack mature mechanisms for joint planning of multiple imaging satellites when dealing with moving target loss. Consequently, with limited a priori information on lost targets, achieving rapid search operations for targets in large sea areas has become a critical challenge in the field of multi-satellite imaging planning.

This study addresses the problem of rapid search mission planning for lost moving targets at sea in emergency situations. The moving target search problem involves two main aspects: predicting the potential area of the moving target and scheduling the imaging satellite mission for search operations [7]. However, due to the limitations of timeliness and the complexity of prediction, the model is only applicable to the trajectory prediction of moving targets at sea under the ideal state. Rajamanickam [8] adopted the Kalman filtering algorithm to predict the next possible position of the target by using the Kalman filter, which can track the target in a simple and real-time way and improve tracking efficiency. However, the filtering method has poor applicability to sea surface motion in complex situations. To solve the problem of complex target distribution, target distribution prediction based on the Markov and Gaussian models has become a research hotspot in recent years. Qiao [9] proposed an adaptive parameter selection trajectory prediction algorithm with the hidden Markov model (HMM) to address the problem of trajectory prediction algorithms in obtaining the positions and behaviors of the moving objects in grid-constrained environments. The HMM can change the objects according to the dynamic change in speed to capture the parameters required for realistic scenarios and achieve efficient trajectory prediction. Dalsnes [10] used a Gaussian mixture model to predict the ship’s position in the following 5–15 min, introducing a measure of uncertainty. Rong [11] decomposed ship motion prediction into longitudinal and transverse directions and predicted trajectory uncertainty based on a Gaussian process model in real time to achieve a better prediction result. Although this class of methods improves the trajectory prediction accuracy, the a posteriori nature of the Markov model and the dependence of the Gaussian process model on the initial state prevent them from incorporating limited a priori information into the model.

The imaging satellite mission scheduling search problem has been a research hotspot in the field. For the daily scheduling problem of SPOT5, Gaberl [12] adopted the related graph theory to transform it into a directed weighted acyclic graph and establish an integer planning model. For the multi-satellite mission planning problem of point targets, Globus [13] compared the performance of the simulated annealing algorithm, hill climbing algorithm, genetic algorithm, and other solution algorithms through simulation experiments, but did not consider constraints such as downlinking data from the satellites and the limitation of storage capacity. Li [14] investigated the mechanism of joint search using electronic reconnaissance satellites and imaging satellites for ocean targets. Wen [15] used constraint-satisfying genetic algorithms to plan the observation of moving targets after predicting the target’s future trajectory and positional information through neural network algorithms. Chen [16] configured evolutionary parameters through deep reinforcement learning and used four satellites to image targets in ground areas. Although extensive research has been conducted on the imaging satellite mission scheduling problem, most of the current imaging satellite mission scheduling searches are based on the search for point targets or regional stationary targets, and the search targets or regions are assumed to be in a stationary state. However, when there is a paucity of a priori information about the target, the possible motion range of the target constitutes a potential motion region, and the range of the potential region varies continuously with the time parameters. Therefore, the fast search mission scheduling of imaging satellites for dynamically variable potential regions is a current research challenge.

Based on the shortcomings of the existing research, this article investigates the moving target potential region prediction problem and the imaging satellite mission scheduling search problem in a specific time-limited mission scenario to optimize the search time and efficiency. Specifically, this study focuses on the following issues:

(1) The construction of a composite moving target prediction method that fuses distance and heading. This study aims to construct a composite moving target prediction method that can fuse distance and heading information. The method will combine a uniform distribution model and a Gaussian distribution model for predicting the distance and heading of a target, respectively, and calculate the instantaneous distribution probability of the target through Bayes’ theorem. In addition, the study will explore how to effectively incorporate uncertain prior information into motion modeling to exploit the limited prior knowledge to predict the possible distribution probabilities of the target.
(2) The establishment of an area search scheduling model based on the maximum probability of overlap strategy. In this study, a regional search and scheduling model based on the “maximum overlap probability” strategy is developed. The model must consider the limitations of the satellite over the top time window, dynamically adjust the target potential region, and take the overlap probability of the target field of view as the optimization objective function. The core of the research lies in improving the search efficiency and performance of the search task through reasonable model design, to ensure that the optimal search effect is realized under limited resources.
(3) The construction of an improved adaptive immunogenetic optimization model. The model aims to significantly improve the optimization performance of the algorithm by dynamically adjusting the optimization search parameters. The study will introduce the diversity and self-regulation characteristics in the immune mechanism so that the algorithm can automatically adapt to different problem characteristics during the search process, thus improving the success rate of the search. At the same time, the study will also explore how to significantly improve the search efficiency by optimizing the parameter settings of the model to ensure more accurate search results in a limited time.

## 2. Description of the Problem

In this study, we consider a lost target search scenario at sea that contains uncertain a priori information. An electronic satellite detects the position of a vessel at a certain moment, after which the target vessel is silenced due to the failure of communication means or the switching on of electromagnetic silence. The position is defined as the a priori point of the target, and the corresponding moment is the a priori moment; the a priori speed, a priori heading, and other information can be obtained. Set the start time of the search task as $T_{e}$ and the end time as $T_{s}$ according to the fast search requirements in this study:

(1) $$T_{s}-T_{e}\leq 2h$$

According to the pre-prediction as well as experience, the speed and heading of the target vessel will vary within a certain range, thus enabling the construction of a collection of target potential areas based on the satellite overtopping moment $R_{s}=\left[R_{T_{1}},R_{T_{2}},\cdots ,R_{T_{N}}\right]$. After the ground system confirms that the target is lost, the low-orbit imaging satellite receives the search command and observes the target potential area in order to regain the target position information. To facilitate the processing of the problem, drawing on the region processing method of Zhang Hailong [17], the region R is divided into N grids, and the set of grid center points $N_{R}$ is constructed. The imaging satellite observes the region denoted by $N_{R}$ at the moment T from the space orbit using the on-board remote sensors and searches for the target in the region at the moment T.

The remote sensors on the $N_{s}$ imaging satellites are generally able to side-swing during observations, and there are several selectable strips within the angle of the sideways swing: $Strip^{T}=\left\{Strip_{1}^{T},Strip_{2}^{T},\cdots ,Strip_{k}^{T}\right\}$ indicates the k set of selectable observation strips when the satellite is in transit at the time of T. The satellite can select only one strip from $Strip^{T}$ for observation according to the advanced search strategy to conduct a fast and efficient search for sea surface targets. The scenario is described as follows.

There are $N_{s}$ imaging satellites observing the potential region $R_{T_{N}}$ constructed at a given time $\left[T_{e},T_{s}\right]$ corresponding to the overtopping moment $T_{N}$. The target motion is predicted based on the target position and velocity a priori information, and the target probability distribution is given for that moment. When the satellite passes over $R_{T_{N}}$, it selects a strip from its collection of observation strips according to the search strategy formulated in the previous stage and conducts observation until it finds the specified target, and the task is completed.

## 3. Moving Target Potential Region Construction and Distribution Probability Modeling

### 3.1. Construction of Target Potential Areas

We define the range of the area where the moving target may appear at $T_{N}$ as the potential area of the target; because the target is in constant motion, the potential area of the target changes at different moments [18].

**Figure 1:**
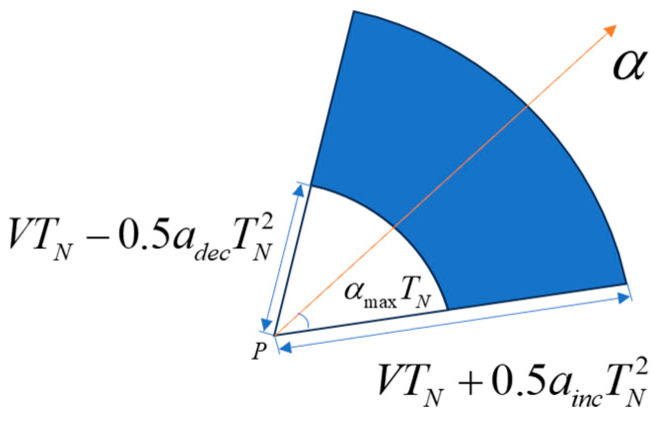
Schematic representation of potential areas.

Assuming that the a priori information of the known target is the a priori position P, the a priori speed V, the a priori heading angle α, the maximum value of speed change $V_{\max}$, the maximum value of heading change $\alpha_{\max}$, the maximum acceleration $a_{inc}$, and the maximum deceleration $a_{dec}$, the potential area of the target $T_{N}$ at any time is calculated as follows: take the Gaussian projection of the a priori position P as the center of the circle drawn with $VT_{N}+0.5a_{inc}T_{N}^{2}$ and $VT_{N}-0.5a_{dec}T_{N}^{2}$ as the radius, take two arcs with the range of the heading angle change $\left[\alpha -\alpha_{\max}T_{N},\alpha +\alpha_{\max}T_{N}\right]$, and set the sector ring area between the arcs as the potential area. The heading angle is defined as the angle between the moving direction and the due east direction, where counterclockwise is positive. Limit $(\alpha +\alpha_{\max}T_{N})-(\alpha -\alpha_{\max}T_{N})\leq 360^{\circ }$. The potential region is shown in Figure 1.

For ease of calculation, the four vertex coordinates of the sector ring were converted to the geodetic coordinate system, and a trapezoidal region was constructed for the calculation. The error relative to the satellite reconnaissance field-of-view range is negligible.

### 3.2. Modeling of the Target Probability Distribution

The target transfer probability distribution is jointly determined by the target’s distance transfer probability distribution at the point and the heading transfer probability distribution. For the distance transfer probability distribution, a uniform distribution model is used to establish the transfer probability density function of the movement distance, and the target’s linear movement distance is uniformly and randomly distributed in its value domain, expressed as

(2) $$△l(t_{1},t_{2})\sim U(△l_{\min},△l_{\max})$$

The range of values $\left[△l_{\min},△l_{\max}\right]$ are discussed in separate cases. In the first time period $\left[0,T_{1}\right]$, the initial velocity of the target is $V_{i}$, and the average velocity of the target is uniformly distributed between $\left[V_{i}-a_{dec}△t,V_{i}+a_{inc}△t\right](△t=T_{1})$; while for the subsequent time period $\left[T_{i},T_{i+1}\right](i\geq 1)$, the initial velocity of the target is $v_{i}$, which is the instantaneous velocity at the moment of $T_{i}$. Then, the range of target motion distance values can be expressed as

(3) $$\{\begin{array}{c} △l_{\min}=v_{i}△t-0.5a_{dec}△t^{2}\\ △l_{\max}=v_{i}△t+0.5a_{inc}△t^{2} \end{array}$$

where $△t=T_{i+1}-T_{i}$, $v_{i}$ is approximated as the ratio of target displacement to sailing time at that moment, i.e., $v_{i}=△l(T_{i})/T_{i}$.

For the heading transfer probability distribution, a Gaussian distribution is used to establish the transfer probability density function for the heading angle. It is assumed that the probability of the target moving along the heading at the starting moment $T_{i}$ during the prediction within $\left[T_{i},T_{i+1}\right](i\geq 0,T_{0}=0)$ is maximized, i.e., $f(\theta_{i})\sim N(\alpha_{T_{i}},\sigma_{\alpha }^{2})$. This is denoted as

(4) $$f(\theta_{i})=\frac{1}{\sqrt{2\pi }\sigma_{\alpha }}e^{-\frac{{\left(\theta -\alpha_{T_{i}}\right)}^{2}}{2\sigma_{\alpha }^{2}}}$$

**Figure 2:**
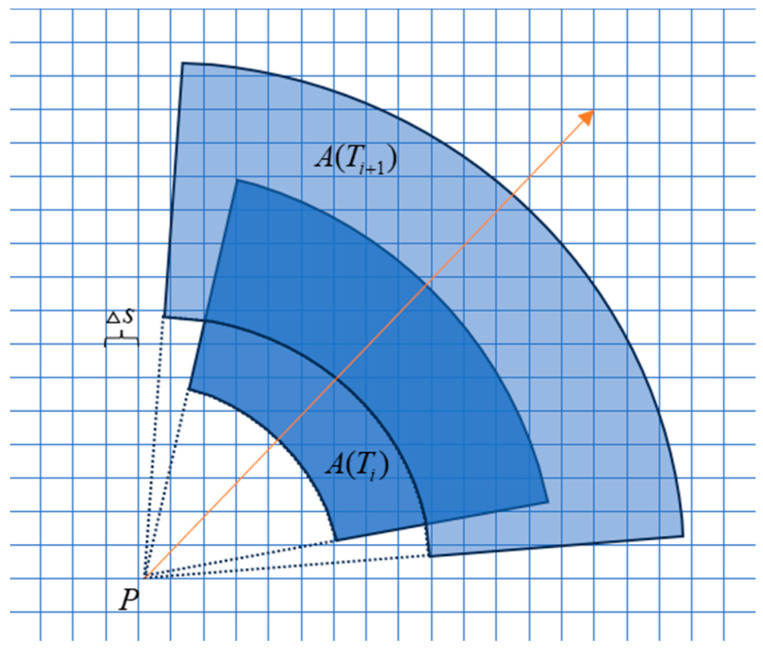
Potential regions at different moments in time.

According to the principle of 3σ and stipulating $\sigma_{\alpha }=\alpha_{\max}/3$, the target’s heading is distributed within $\left[\alpha_{T_{i}}-\alpha_{\max},\alpha_{T_{i}}+\alpha_{\max}\right]$ with a probability of 0.997. Since the starting moment heading $\alpha_{T_{i}}$ changes during $\left[T_{i},T_{i+1}\right](i\geq 1)$ prediction, this article proposes a method for $\alpha_{T_{i}}$ prediction using autoregressive models for each time point of $T_{i}>T_{0}$, assuming

(5) $$\alpha_{T_{i}}=\alpha_{T_{i-1}}+\varepsilon_{T_{i}}$$

where $\varepsilon_{T_{i}}$ is a random error term drawn from $f(\theta_{i})$. At each time point, the predicted value from the previous time point is summed with a new random perturbation term to obtain the predicted value from $\alpha_{T_{i}}$ at the current time point. The size of the potential region at different moments in time is shown in Figure 2.

Due to the high time sensitivity of the fast search task scenario, the distance transfer probability distribution and the heading transfer probability distribution can be assumed to be independent of each other. Then, the transfer probability density distribution of the target in the task scenario can be expressed as

(6) $$f(△l,\theta_{i})=p(△l)•f(\theta_{i})$$

Since the region R is gridded, the grid transfer probability distribution can be approximated as the transfer probability distribution between the points in the grid with the grid granularity △s. Let $P_{m,n}(T_{i},T_{i+1})$ denote the transfer probability that the target $T_{i}$ is located in the grid m at the time and transferred to the grid at the time $T_{i+1}$. Then, the following integral can be obtained:

(7) $$P_{m,n}(T_{i},T_{i+1})=\int_{\lambda_{n}-\frac{1}{2}△s}^{\lambda_{n}+\frac{1}{2}△s}\int_{\varphi_{n}-\frac{1}{2}△s}^{\varphi_{n}+\frac{1}{2}△s}f[l_{m},(\lambda ,\varphi )]d\lambda d\varphi$$

where $l_{m}$ are the coordinates of the center point of the grid m, and λ and φ denote the geographical latitude and longitude at any moment, traversing the set of all grid distribution combinations to obtain the transfer probability distribution of the target in that time period.

The transfer probability is essentially a conditional probability, indicating the probability of transferring to n at the next moment under the condition that the target is located at m. Assuming that the potential region is divided into $N_{k}$ grids at the current moment, let $P_{n}(T_{i+1})$ denote the instantaneous probability that the target is located in the grid n at the moment $T_{i+1}$, which can be obtained using Bayes’ rule, P(n)=P(n|m)P(m):

(8) $$P_{n}(T_{i+1})=\sum_{m=1}^{N_{k}}P_{m,n}(T_{i},T_{i+1})•P_{m}(T_{i})$$

Therefore, the instantaneous probability distribution of the target in the potential region at the current moment is obtained iteratively from the a priori information that the target is located at point P at moment $T_{0}$.

## 4. Satellite Search Scheduling Model Based on the IAIGA

### 4.1. Definition of Model Elements

Since the potential area R was gridded at the grid granularity △s during the satellite transit, a total of m grids were included in the potential area. The assumption of boundary grid coverage is made here: when the satellite’s field of view covers the potential area grids, a grid is considered to be observed by the satellite when its center and at least two vertices are within the field of view.

### 4.2. Objective Function

Since the target is in real-time motion, the potential area is constantly changing during each satellite transit. The static domain assumption is made here: let the state of the search region at the midpoint time of the time window be the state of the whole time window; the potential area and probability distribution of the target will not change during the transit of the satellite in that time window. Let the midpoint time of the time window be $T_{i}$, and to facilitate the representation of the region $Strip_{jk}^{T_{i}}$, the following one-dimensional vector is defined:

(9) $$X_{i}=\left[x_{i,1},x_{i,2},\cdots ,x_{i,N}\right]$$

The vector represents the coverage of the search domain $R_{i}$ by the ith observation strip, where $N=N_{m}(T_{i})$ denotes the total number of grids where this observation strip region $Strip_{jk}^{T_{i}}$ coincides with the potential region $R_{i}$, and m denotes the mth grid, and the elements of the vector take the following values:

(10) $$x_{i,m}=\{\begin{array}{l} \begin{array}{cc} 1 & {\;\;\mathrm{Grid}\;m\;\mathrm{is}\;\mathrm{in}\;\mathrm{the}\;\mathrm{region}\;R}_{i} \end{array}\\ \begin{array}{cc} 0 & {\mathrm{Grid}\;m\;\mathrm{is}\;\mathrm{not}\;\mathrm{in}\;\mathrm{the}\;\mathrm{region}\;R}_{i} \end{array} \end{array}$$

**Figure 3:**
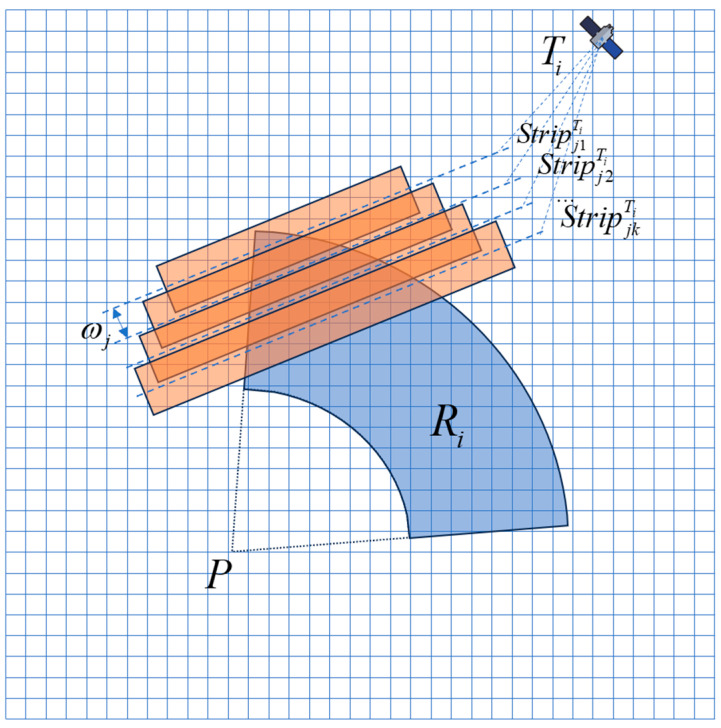
Satellite candidate action set.

The collection of satellite candidate actions is shown in Figure 3.

Then, the total probability that the satellite’s field of view coincides with the grid of potential regions within the observation strip $Strip_{jk}^{T_{i}}$ is

(11) $$P_{i}(Strip_{jk}^{T_{i}})=\sum_{m=1}^{N}x_{i,m}P_{m}(T_{i})$$

The optimization objective of the model is to plan each satellite for strip selection to maximize the total probability of the target discovery sum. This is denoted as

(12) $$Strip^{T_{i}}(Selected)=\underset{k}{\max}\sum_{j=1}^{N_{s}}\sum_{i\in Strip_{j,k}^{T}}\left(P_{i}(Strip_{jk}^{T_{i}})\right)$$

### 4.3. Constraints

Satellite observation mission planning requires careful consideration of various constraints, including satellite orbit, attitude control capability, payload performance, and environmental factors. The time constraint mandates that the observation mission be conducted within the satellite’s window of visibility to the potential region. The space constraint primarily refers to limitations in the satellite’s observation field of view, which are influenced by the configurations of the payloads carried by the satellite. In fast search missions, due to the requirement for a short total mission duration, the satellite can often only transit the target area once, or sometimes not at all. These time constraints can be addressed before mission planning, effectively eliminating them from consideration. The main constraints are summarized as follows:

(1) $T_{i,s}-T_{i,e}\leq d_{i,j}$, satellites search for observations in a time window that is less than the maximum power-on time of the satellite’s remote sensors.
(2) $A_{i,j}\leq A_{j}$, indicating that the remote sensing element side-swing angle is less than the maximum side-swing angle when the satellite performs its mission in a time window.
(3) $w_{i_{1},j_{1}}y_{i_{1},j_{1}}+w_{i_{2},j_{2}}y_{i_{2},j_{2}}\leq 1$, where $w_{i_{1},j_{1}},w_{i_{2},j_{2}}$ performs normalization to indicate the selection of a task for planning when the observation task time windows overlap.
(4) $me_{i,j}\leq M_{j},\forall i\in T,j\in S$, which indicates the storage capacity limit at any given moment.
(5) $M_{j}^{st}=M_{j}^{ed}=M_{j},\forall j\in S$, indicating that the satellite storage capacity begins and ends with the maximum storage capacity.

### 4.4. Improvement of Adaptive Immunogenetic Algorithms

The immunogenetic algorithm (IAIGA) is a population-based heuristic optimization algorithm that combines the principles of immunity theory with a basic genetic algorithm. While genetic algorithms are highly robust in addressing optimization problems, they often encounter issues of falling into local optima. The IAIGA enhances this by automatically adjusting the algorithm’s parameters and strategies to adapt to the specific characteristics of different problems, mimicking the adaptive traits of the biological immune system. Additionally, the algorithm employs a diversity preservation mechanism inspired by the immunity principle, effectively avoiding premature convergence and local optima. This significantly improves the algorithm’s global search capability.

(1) Coding

The decision variable of the model is the selection of satellite observation strips, i.e., $Strip_{R_{i}}^{s}=\left\{Strip_{R_{i_{1}}}^{s_{1},n_{1}},Strip_{R_{i_{2}}}^{s_{2},n_{2}},\cdots ,Strip_{R_{in}}^{s_{N_{s}},n_{m}}\right\}$, where $Strip_{R_{i}}^{s_{N_{s}},n_{m}}$ indicates that the satellite $s_{N_{s}}$ selects the $n_{m}$th pendulum angle when transiting the region $R_{i_{n}}$. According to the set of different observation strips composed by different satellites, $Strip_{R_{i}}^{s}$ can be regarded as a chromosome, and $Strip_{R_{i}}^{s_{N_{s}},n_{m}}$ can be regarded as a gene.

(2) Adaptation function

The objective function of the fast search task is taken as the fitness function, i.e.:

(13) $$Strip^{T_{i}}(Selected)=\underset{k}{\max}\sum_{j=1}^{N_{s}}\sum_{i\in Strip_{j,k}^{T}}\sum_{m=1}^{N}x_{i,m}P_{m}(T_{i})$$

(3) Algorithm flow

Step 1: Population initialization. Randomly generate a set of initial populations, with each individual corresponding to a solution in the solution space. The initial populations should be diverse to cover various regions of the solution space.

Step 2: Fitness assessment. Evaluate the degree of superiority or inferiority of an individual in the solution space according to the fitness function.

Step 3: Roulette method for next-generation selection. Based on the fitness value of individuals, a group of the best individuals is selected for the next generation. The probability of an individual being selected is shown in Equation (14).

(14) $$p(x_{i})=\frac{F(x_{i})}{\sum_{j=1}^{N}F(x_{j})}$$

Step 4: Crossover operation. The crossover operation is performed on the selected individuals, i.e., two individuals are randomly selected for gene exchange according to a certain crossover probability $P_{c}$.

Step 5: Mutation operation. A mutation operation is performed on the crossed individuals, i.e., some genes of the individuals are randomly changed according to a certain mutation probability $P_{m}$.

Step 6: Immunization operation. The immunological selection of individuals is performed based on their fitness values and concentrations. Immunological selection can retain individuals with higher fitness and some diversity. When calculating the antibody concentration, the antibody affinity can be calculated first, and the affinity between the two reflects the degree of similarity between the antibodies.

The affinity was calculated as in Equation (15):

(15) $$S_{v,s}=\frac{k_{v,s}}{L}$$

where $k_{v,s}$ is the number of bits of the same code in the two sets of antibody v and antibody s, and L is the length of the antibody.

Antibody concentration $C_{v}$ is the proportion of similar antibodies in the population to the total population and is calculated as

(16) $$C_{v}=\frac{1}{N}\sum_{j\in N}S_{v,s},S_{v,s}=\{\begin{array}{l} 1,S_{v,s}>T\\ 0,\mathrm{else} \end{array}$$

where N is the total number of antibodies and T is the similarity threshold.

Step 7: Iterative update. After selection, crossover, mutation, and immunization operations, the individuals are formed into a new population and undergo Step 2 for the next round of fitness assessment and selection operations until the termination condition is met.

Since the potential region and candidate actions change during the fast search process of moving targets, the algorithm is improved with an adaptive strategy to ensure the convergence speed and stability while ensuring the accuracy of the search. In the process of the immune algorithm, the crossover probability $P_{c}$ and the variation probability $P_{m}$ make it difficult to find a fixed optimal value, and the size of their values will affect the convergence speed of the immune genetic algorithm. When the individuals in the population converge, the values of $P_{c}$ and $P_{m}$ should be adjusted to be larger, and when the fitness is more dispersed, the values of $P_{c}$ and $P_{m}$ should be adjusted to be smaller. The adjustment method is shown in the following equation:

(17) $$P_{c}=\{\begin{array}{l} \begin{array}{cc} k_{1}-\frac{\left(k_{1}-k_{2})(f^{\prime }-f_{avg}\right)}{\left(f_{\max}-f_{avg}\right)} & ,f^{\prime }\geq f_{avg} \end{array}\\ \begin{array}{cc} k_{1} & \;,f^{\prime }<f_{avg} \end{array} \end{array}$$

(18) $$P_{m}=\{\begin{array}{l} \begin{array}{cc} k_{3}-\frac{\left(k_{3}-k_{4})(f^{\prime }-f_{avg}\right)}{\left(f_{\max}-f_{avg}\right)} & ,f\geq f_{avg} \end{array}\\ \begin{array}{cc} k_{3} & \;,f<f_{avg} \end{array} \end{array}$$

where $f_{\max}$ and $f_{avg}$ are the maximum fitness value in the population and the average fitness value of the population in each generation, respectively; $f^{\prime }$ is the fitness value of the larger of the two individuals to be crossed; f is the fitness value of the individual to be varied; and $k_{1}$,$k_{2}$,$k_{3}$,and $k_{4}$ are all adaptive control parameters that take fixed values in the [0,1] interval.

**Figure 4:**
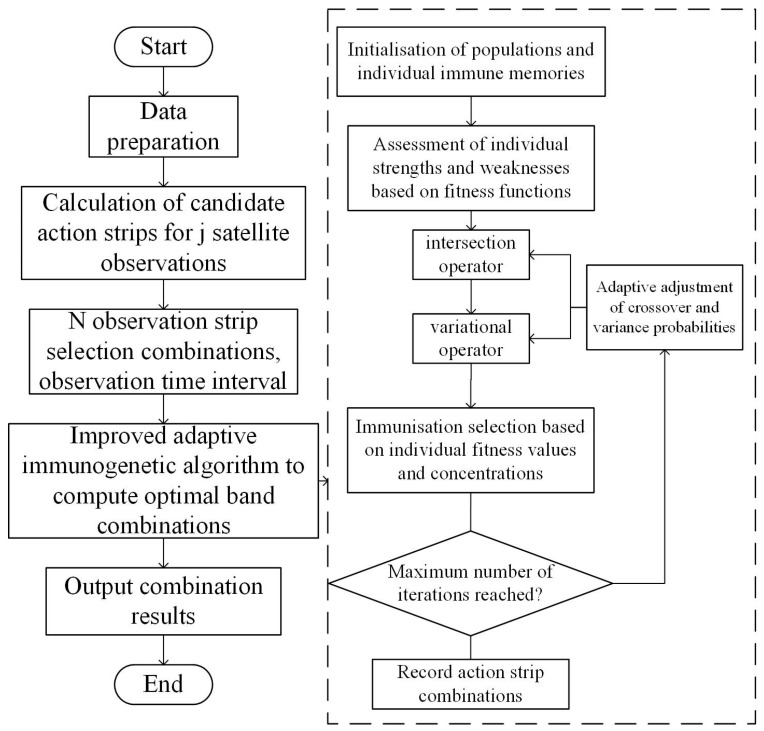
Improved adaptive immunogenetic algorithm.

The IAIGA is shown in Figure 4.

## 5. Simulation Experiments and Results

### 5.1. Scene Parameter Configuration and Visible Window Calculation

Simulation experiments were conducted for the lost moving target search at sea in emergency situations. The specific scenario of the experiment was set as follows: a large cargo ship is lost in the Pacific Ocean, and its last position information is 08:00:00.000 on 18 April 2024, located at [11°N, 120°E], with a speed of 18 knots/nautical miles, and the heading angle is 20°. According to the preliminary prognosis, the maximum yaw angle after the vessel was lost was 10°, the maximum acceleration was 5 km/h^2^, and the maximum deceleration was 2 km/h^2^. After discovering that the vessel was lost, the emergency plan was immediately activated, and low-orbit imaging satellites were used to observe the relevant sea area and regain the position information of the target. Due to the need to quickly search for the ship’s position, the moment of annotation to the satellite on the command was set as 19 April 2024, 12:00:00.000, and the mission deadline as 19 April 2024, 14:00:00.000, and the satellite searched the potential area within two hours after receiving the command. The available imaging satellite resources included six low-orbit optical imaging satellites selected from domestic and foreign civilian optical imaging satellites, whose specific parameters are shown in Table 1.

The observation capabilities of the satellite platforms selected for the experiment are shown in Table 2.

Before scheduling optimization, it was necessary to calculate the satellite’s window of visibility to the potential search domain at the moment of overtopping, based on the geometrical relationship between the star–earth space and the orbital model. Considering the actual operational conditions of satellites, only one satellite can perform an observation task within a given time window. If there is a conflict in time windows, the satellite with a higher probability of discovery in the overlapping area between its field of view and the potential region will be selected to carry out the observation task. The window information is presented in Table 3.

**Figure 5:**
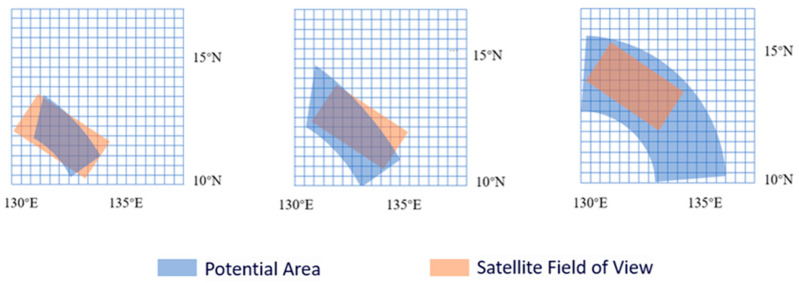
Satellite transit observable area and search domain dispersion.

The observable area of the satellite transit and the discretization of the search domain are shown in Figure 5.

### 5.2. Trajectory Set Observation Test Validation

**Figure 6:**
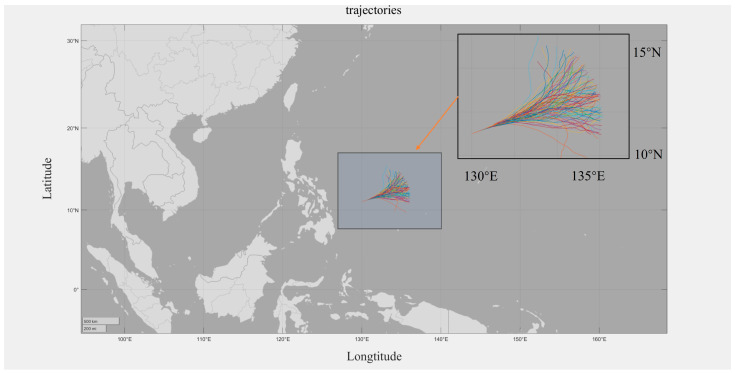
Standardized trajectory set.

In order to perform simulation calculations and evaluate the task scenarios set out in Section 4.1, the trajectory set targeting method was used to test and analyze the scenarios. The standard ship trajectory set was used for testing, and one set containing 100 possible ship trajectories was randomly generated according to the target heading range restriction and speed range restriction. The standard trajectory set visualization is shown in Figure 6.

#### 5.2.1. Feasibility Analysis of the Maximum Probability of Overlap Optimization Metrics

**Figure 7:**
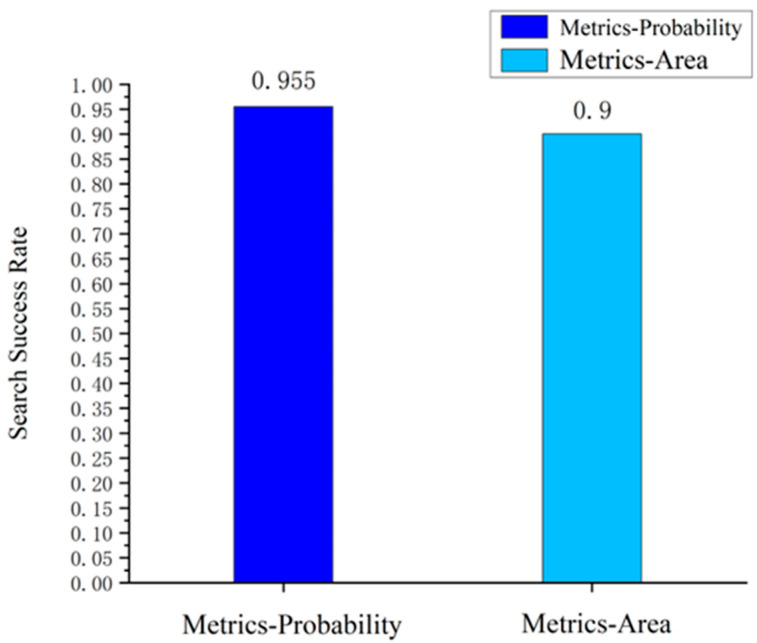
Comparison of target shooting results with different objective functions.

In the past, the “maximum search area” was usually used as a common optimization index in the satellite search scheduling for the region. The maximum search area represents the coverage of the satellite’s field of view over the search area, and in the process of satellite scheduling, the sub-observation zones that can maximize the coverage of the target area grid are selected to improve the coverage of the total satellite observation area. Considering the variability of the target area of the search problem, this study proposes an observation strategy for dynamically variable areas, i.e., establishing an optimization index based on a probabilistic prediction model, aiming to achieve the maximum search probability of the overlap between the satellite sub-observation band’s field of view and the potential target area, i.e., the maximum overlap probability search strategy. Therefore, compared with the coverage ratio, the applicability of the search probability index to the dynamic target search problem proposed in this article was the first issue to be considered. The traditional maximum search area and maximum search probability were taken as the objective functions, and the statistics of the targeting results obtained using the IAIGA are shown in Figure 7.

As shown in Figure 7, under the premise of using the IAIGA for optimization, the targeting results based on the maximum probability of the overlap optimization index were significantly better than the targeting results based on the maximum coverage area optimization index. The satellite search success rate based on the maximum overlap probability optimization index was about 6% higher than that of the maximum coverage area optimization index, which can effectively search for lost targets in the dynamically variable potential area.

#### 5.2.2. Feasibility Analysis of the “Prediction-Scheduling” Search Strategy

**Figure 8:**
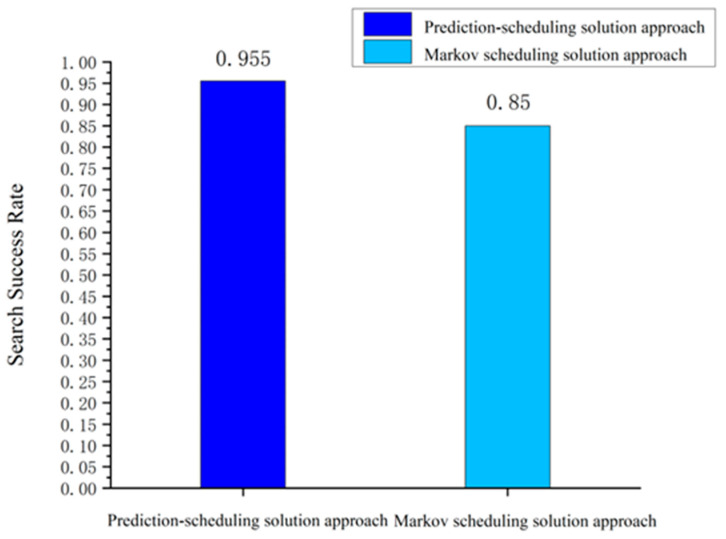
Comparison of target shooting results for scheduling methods.

In previous studies, Kalman filtering and Markov chain methods have usually been used to model the motion of a moving target to simulate this motion when the target trajectory is unknown and lost. These types of methods can quickly model the probability density function of the target in the potential area and use it as an indicator to observe the potential target area. However, the limitation of such methods is that they cannot fully utilize the limited a priori information of the target as a guide for prediction. In this article, we propose the method of composite heading transfer probability distribution and distance transfer probability distribution to obtain the prediction results of the probability distribution of the target in the potential target area and use this method to guide satellite search scheduling. The results of comparing the success rate of the Markov prediction-scheduling method with the prediction-scheduling method proposed in this article are shown in Figure 8.

It can be seen that using the prediction-scheduling search strategy proposed in this article for a sea-surface moving target search resulted in a significant improvement in the search success rate, reaching an increase of 12.3% compared to the Markovian prediction-scheduling approach. This fully demonstrates the advantages of the strategy in coping with the problem of a lost moving target search at sea in emergency situations.

In addition, given the target information, mission requirements, and imaging satellites, the temporal distribution of the observation window as well as the transit observable range are fixed, which leads to specific scenarios where the target cannot be searched for no matter how the scheduling is conducted. To avoid the influence of the search condition limitations on the validation of the search strategies, a judgment index is defined—the search success rate relative to the optimal solution of the trajectory set. To determine the optimal observation scheme using the exhaustive enumeration method, i.e., for a certain track set $S_{i}$, by comprehensively searching for all possible combinations of observation directions and observation periods of all satellites, we find the set of satellite observation strips with the highest search success rate for this track set, i.e., the optimal scheme $B\mathrm{est}(S_{i})$ for this track set. Let $N_{Plan}(S_{i})$ be the number of trajectories successfully searched in the $S_{i}$ set using the search scheme obtained via the prediction-scheduling method proposed in this article, and $N_{Best(i)}(S_{i})$ be the number of trajectories successfully searched in the $S_{i}$ set by the optimal scheme. Then, the search success rate of the optimal scheme $B\mathrm{est}(S_{i})$ for the track set is defined as

(19) $$SR(S_{i})=\frac{N_{Plan}(S_{i})}{N_{Best}(S_{i})}$$

**Figure 9:**
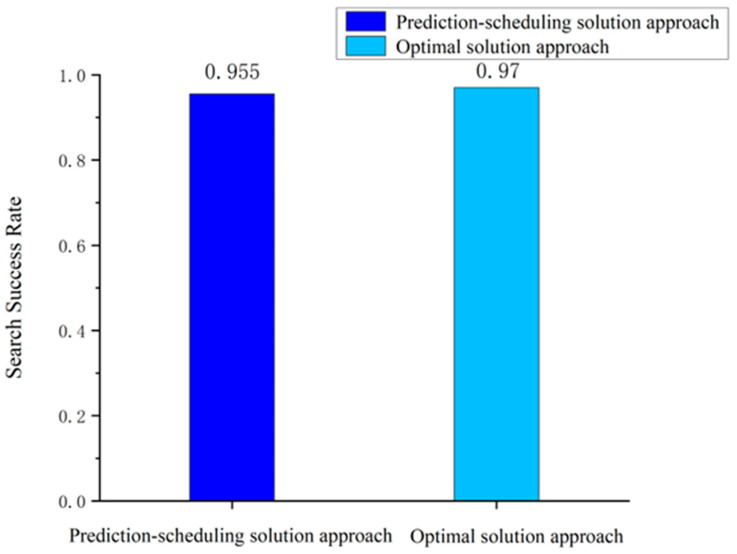
Comparison of search success rates between the optimal solution and the prediction-scheduling search solution.

A comparison of the search success rate of the optimal solution with that of the prediction-scheduling search solution is shown in Figure 9.

Since the goal of the optimal search scheme was to traverse the set of all possible candidate actions and identify the scheme with the highest search success rate, the search success rate of the optimal search scheme was slightly higher than that of the prediction-scheduling method. According to Equation (19), the search success rate of the prediction-scheduling method with respect to the optimal program of the trajectory set was 98.4%. The prediction-scheduling search scheme proposed in this article had better applicability to the satellite search scheduling in the dynamic variable region.

### 5.3. Scheduling Algorithm Effectiveness Analysis and Comparison

**Figure 10:**
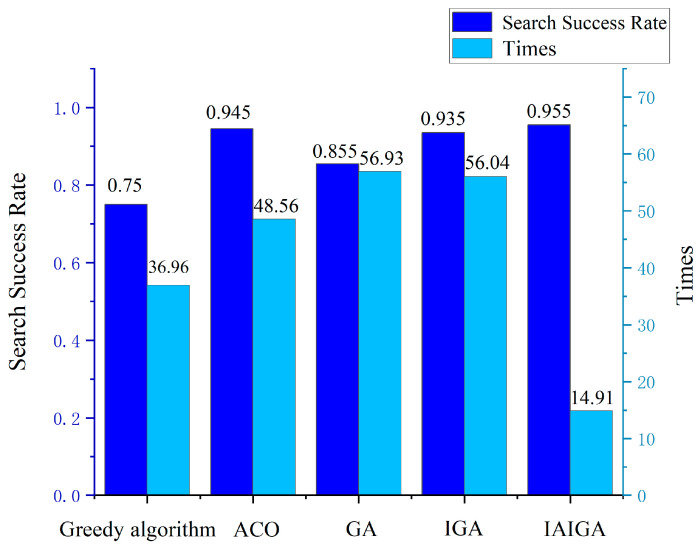
Comparison of search success rate and algorithmic solution time.

The feasibility analysis of the optimization index in Section 5.2.1 determined the search strategy based on maximum overlap probability as the optimization index. The evaluation indexes of the effectiveness of the scheduling algorithm have two main aspects: the algorithm search success rate and the algorithm solving efficiency. At present, the satellite mission planning field mostly adopts intelligent optimization algorithms for task-scheduling optimization, and the algorithms used for comparison are the greedy algorithm, the ant colony algorithm (ACO), the genetic algorithm (GA), the immunogenetic algorithm (IGA), and the IAIGA proposed in this article. The evaluation indexes are the trajectory search success rate and algorithm solution time. A comparison of the search success rate and algorithm-solving time is shown in Table 4 and Figure 10.

From Figure 10, it can be seen that the greedy algorithm and the GA did not perform well in the complex scenarios of the sea-surface moving target search, and neither of them reached a level over 90%. On the other hand, the ACO, IGA with immunization algorithm, and IAIGA showed higher search success rates, at over 90%. This indicates that global optimization algorithms such as the ACO and the improved genetic algorithm can better adapt to the search for lost targets in dynamic variable regions and can more accurately perform target discovery. As for the comparison of algorithmic solution times, the IAIGA exhibited obvious advantages. Compared with the ACO, the IAIGA reduced the algorithm’s solution time by about 70%. Compared with the IGA, the IAIGA reduced the algorithm’s solution time by about 73%, which reflected the excellent solution efficiency of the IAIGA. Through a comprehensive comparison of the two, the IAIGA was found to shorten the solution time and improve the solution efficiency while stabilizing a higher search success rate, and it demonstrated high solution efficiency in the lost moving target search scenario discussed in this article.

## 6. Discussion

### 6.1. Advantages of the IAIGA-Based Predictive-Scheduling Approach

Investigating the search problem of lost moving targets at sea is crucial to addressing issues related to maritime search and rescue operations, as well as to enhancing maritime law enforcement capabilities. Recognizing the limitations of the current research concerning the rapid search problem of imaging satellites for lost moving targets at sea, which tends to be time-consuming and fails to meet the requirements for swift target location, this study considers the dynamic nature of sea movement exhibited by moving targets. To address this, we establish a task planning model by constructing a dynamic potential area search model with an objective function centered on maximizing the probability of overlapping areas. This model was then optimized using the IAIGA. In this paper, we present a dynamic potential area search model, establish a mission planning model with the maximum probability of overlapping areas as the objective function, and address the problem through optimization using the IAIGA. Through experimental validation, we showcase the effectiveness and superiority of the prediction-scheduling search strategy and the IAIGA in the context of rapid search and planning for satellite imaging at sea. In comparison with traditional methods, our approach offers several advantages.

First, the prediction-scheduling search strategy proposed in this article fully integrates the target’s a priori information into the satellite search planning model. Given the characteristics of lost targets, which include limited a priori information and random motion direction [19], establishing a reasonable prediction model to simulate the possible locations of the target is crucial to avoid wasting search resources. To address this problem, we consider both the target heading transfer probability density and the distance transfer probability density in our predictions, establishing a target probability distribution model that incorporates prior information. This approach combines the limited a priori information of the target with the motion model to predict the target’s distribution probability within the potential area. Compared to other motion prediction models [20], our proposed model is better suited for addressing the challenges associated with wide-ranging moving target areas and expanding potential areas. In the feasibility analysis of the model, we included the search success rate relative to the optimal solution of the trajectory set as a judgment indicator. This demonstrates the applicability of the prediction-scheduling method in moving target search scenarios, enhancing the efficiency of satellite scheduling while ensuring a high success rate in satellite search operations.

Second, the evaluation index of the maximum probability of overlap proposed in this article offers better search benefits. Typically, it is challenging for ground targets to evade a satellite’s field of view under visible conditions, as electronic remote sensing satellites, optical imaging satellites, and SAR imaging satellites can jointly observe and locate low-speed moving targets such as ships at sea, which have limited maneuverability. However, the search conditions for lost targets are more stringent [6]. Since the target is lost and cannot emit radio signals, electronic remote sensing satellites are ineffective, leaving only imaging satellites with smaller fields of view for the search. Additionally, the search domain for a lost target is extensive, meaning that the satellite’s field of view will not cover a large area of the potential target zone. Over time, not only does the uncovered area expand, but the target may also move into previously covered areas. Furthermore, using the maximum search area as the optimization target treats all grids in the potential search area as equally valuable for observation, ignoring the a priori motion information of the target that could guide satellite observation. This approach reduces search efficiency and wastes search resources. In contrast, the maximum probability of the overlap optimization index enables focused observation of the potential search area based on predicted target distribution probabilities. This strategy enhances the success rate of search missions by efficiently using limited satellite field-of-view coverage. Therefore, it can be concluded that, compared to the traditional maximum search area optimization index, the maximum probability of overlap index better utilizes search resources and a priori information. It is more suitable for addressing the target search problem in dynamically variable areas.

In addition, the IAIGA model proposed in this article demonstrates robust global search capabilities. Compared with the greedy algorithm [21], ant colony algorithm [22], genetic algorithm [15], and immunogenetic algorithm, which have been widely used in recent years, the IAIGA model’s innovation lies in its introduction of an adaptive mechanism and an immune mechanism. These mechanisms enable the algorithm to dynamically adjust its search strategy during the search process to accommodate changing environments and demands. Especially when dealing with highly dynamic and uncertain problems such as maritime emergency lost target search, the IAIGA model can flexibly address various challenges, significantly improving the algorithm’s performance. Specifically, the IAIGA model’s global search capability allows it to quickly locate targets within a wide search space. In the context of maritime emergency lost moving target searches, this means that the algorithm can efficiently conduct fast searches for sea surface moving targets, thereby increasing the search success rate. Furthermore, because the algorithm can dynamically adjust its search strategy and reduce solution time, it can locate targets more rapidly and enhance search efficiency in complex and changing maritime environments.

### 6.2. Limitations and Possible Improvements

However, this study has certain limitations and shortcomings that must be considered.

Firstly, the heading transfer probability density model and the distance transfer probability density model proposed in this article adopt uniform distribution and Gaussian distribution, respectively. In actual complex maritime scenarios, the dynamics and uncertainty of the maritime environment can lead to unpredictable changes in the target’s motion. The motion trajectory of the lost target may exhibit anomalous behaviors, such as sharp turns and drifting, which are not accounted for in the ideal model. Future research should consider incorporating maritime environment parameters to simulate real target motion more accurately.

Secondly, this study only considers optical imaging satellites. Optical imaging satellites are affected by factors such as sunlight angle and cloud cover. Additionally, by relying on a single type of optical imaging satellite orbit, the universality of the search scenario is limited. In practical applications, multiple types of imaging satellites are often used in concert to enhance search effectiveness. Therefore, future research will explore moving target search strategies that involve multiple orbital planes and satellite types to adapt to various application scenarios.

Finally, in this study, due to the fast search scenario requirements, the scenario time was set shorter, and the satellite time window was limited. This reduces the computational pressure on the optimization algorithm. However, it is uncertain whether the IAIGA can maintain superior performance for multi-objective large-scale and long-duration searches. As the solution space grows rapidly, the algorithm’s optimization speed may slow down. Future research will focus on exploring algorithm optimization for multi-objective large-scale maritime searches to achieve more optimal solutions for locating moving targets on the sea surface.

## 7. Conclusions

In this study, we explored the problem of fast search planning for lost moving targets in maritime emergencies using the improved adaptive immunogenetic algorithm (IAIGA). Addressing the shortcomings of current research on the rapid search problem of maritime lost target imaging satellites, we constructed a dynamic potential area search model, established a task planning model with the maximum probability of overlapping area as the objective function, and optimized the problem using the IAIGA. Through experimental verification, our main conclusions are as follows:

(1) The composite moving target prediction method, based on distance and heading information, effectively integrates uncertain a priori information, resulting in a more accurate and comprehensive probability distribution of the target under conditions of limited prior information.
(2) The prediction-scheduling search strategy, based on overlap probability maximization, significantly improves search efficiency and performance, providing an effective strategy for satellite search tasks.
(3) Optimizing the moving target fast search planning problem using the IAIGA combines adaptive and immune mechanisms, enabling the algorithm to adapt to different problem characteristics and achieve self-optimization during the search process. This approach demonstrates excellent performance in terms of search success rate and algorithmic solving time, offering strong technical support for practical applications.

## Figures and Tables

**Table 1:** Satellite parameters.

Satellite Name	*a*/km	*i*/(°)	Ω/(°)	e	*ω*/(°)	*M*/(°)
Sat1	6775	97.5820	319.4822	0.009	81.1158	311.88
Sat2	7062	98.2298	194.5448	0.002	92.1078	268.03
Sat3	6972	97.8413	204.6933	0.001	111.172	248.95
Sat4	7347	97.9118	201.5720	0.001	25.4327	334.69
Sat5	7229	97.9135	187.3469	0.001	297.53	189.14
Sat6	6814	98.251	147.204	0.002	46.485	172.394

Note: a is the orbital half-length axis, i is the orbital inclination, Ω is the ascending node declination, e is the orbital eccentricity, ω is the perigee amplitude angle, and M is the flat perigee angle.

**Table 2:** Observation capabilities of satellite platforms.

Satellite Name	Remote Sensor Field of View/°	Maximum Power-on Time/s	Maximum Side-Swing Angle/°	Side Pendulum Rate/(°/s)	Side-Swing Stabilization Time/s
Sat1	5	400	30	0.2	200
Sat2	6	450	30	0.2	200
Sat3	7	500	35	0.3	200
Sat4	7	500	40	0.3	150
Sat5	7	550	40	0.4	150
Sat6	7	550	40	0.4	150

**Table 3:** Satellite overhead time window.

Satellite Name	Start Time	Stop Time	Duration
Sat1	7 March 2024 12:09:55.469	7 March 2024 12:20:29.826	634.357
	7 March 2024 13:42:58.892	7 March 2024 13:53:24.988	626.095
Sat2	7 March 2024 12:09:06.152	7 March 2024 12:24:08.762	902.611
	7 March 2024 13:47:48.302	7 March 2024 14:03:09.674	921.372
Sat3	7 March 2024 12:00:41.158	7 March 2024 12:13:24.359	763.201
	7 March 2024 13:36:46.173	7 March 2024 13:51:00.418	854.246
Sat4	7 March 2024 12:37:44.446	7 March 2024 12:56:18.582	1114.136
Sat5	7 March 2024 13:02:10.258	7 March 2024 13:19:49.275	1059.018
Sat6	7 March 2024 12:22:27.887	7 March 2024 12:33:26.289	658.402
	7 March 2024 13:55:28.136	7 March 2024 14:05:57.235	629.100

**Table 4:** Comparison of search success rate and algorithmic solution time.

Algorithm	Search Success Rate	Time
Greedy algorithm	0.75	36.96
ACO	0.945	48.56
GA	0.855	56.93
IGA	0.935	56.04
IAIGA	0.955	14.91

## Data Availability

Data are available on request from the authors.

## Abbreviations

**Model Elements**	**Definition**
S	Satellite resource set, $S=\left\{s_{1},s_{2},\cdots ,s_{N_{s}}\right\}$, $N_{s}$ is the number of satellites
$FOV_{j}$	Field of view of the satellite $s_{j}$
$A_{j}$	Maximum lateral swing angle of the satellite $s_{j}$
$\omega_{j}$	Side-swing granularity of the satellite $s_{j}$
$d_{j}$	Maximum power-on time of the remote sensors of the satellite $s_{j}$
$y_{i,j}$	Observation maneuvers during one transit of satellite $s_{j}$
$M_{j}$	Storage capacity of the satellite $s_{j}$
$M_{j}^{st}$	Storage capacity at start of the satellite $s_{j}$
$M_{j}^{ed}$	Storage capacity at the end of the satellite $s_{j}$
$T_{e}$	Search mission start time
$T_{s}$	Search mission end time
$R_{s}$	The set of potential regions,$R_{s}=\left[R_{T_{1}},R_{T_{2}},\cdots ,R_{T_{N}}\right]$
$w_{ij}$	ith time window of the satellite $s_{j}$
$Strip_{j}^{T}$	Single-satellite transit candidate action strips, $Strip_{j}^{T}=\left\{Strip_{j1}^{T},Strip_{j2}^{T},\cdots ,Strip_{jk}^{T}\right\}$
